# Changes in phytochemical properties and water use efficiency of peppermint (*Mentha piperita* L.) using superabsorbent polymer under drought stress

**DOI:** 10.1038/s41598-023-49452-z

**Published:** 2023-12-11

**Authors:** Saloome Sepehri, Sima Abdoli, Behnam Asgari Lajayer, Tess Astatkie, G. W. Price

**Affiliations:** 1 Agricultural Engineering Research Institute (AERI), Agricultural Research, Education and Extension Organization (AREEO), P.O. Box 31585-845, Karaj, Iran; 2 Department of Soil Science and Engineering, Shahid Chamran University of Ahvaz, Ahvaz, 6135743136 Iran; 3 Faculty of Agriculture, Dalhousie University, Truro, NS B2N 5E3 Canada

**Keywords:** Plant physiology, Plant stress responses, Secondary metabolism

## Abstract

Water consumption management and the application of advanced techniques in the agricultural sector can significantly contribute to the efficient utilization of limited water resources. This can be achieved by improving soil texture, increasing water retention, reducing erosion, and enhancing seedling germination through the use of superabsorbent polymers. This study aimed to investigate the effect of Aquasource superabsorbent (AS) on the morphological characteristics, phytochemical properties, antioxidant content, and water use efficiency of peppermint. It was conducted under different irrigation management and using different superabsorbent levels. Therefore, a 3 × 4 factorial design was used to determine the effects of irrigation intervals (2-, 4-, and 6-day) and different levels of AS amount (zero [control], 0.5, 1, and 2 wt%). The effects of these factors on various parameters (morphological characteristics, essential oil percentage, nutrient, protein, proline, carotenoid, antioxidant, and chlorophyll content, leaf area index, relative water content, and water use efficiency [WUE]) were evaluated. The results showed that morphological characteristics and essential oil percentage decreased significantly under drought stress (increasing the irrigation intervals). However, the addition of 0.5 (wt%) AS improved plant growth conditions. Increasing the amount of superabsorbent used to 1 and 2 (wt%) decreased the measured traits, which indicates the creation of unsuitable conditions for plant growth. AS application improved the growth of the root more than the leaf yield of peppermint. A 0.5 (wt%) addition of AS resulted in root length increases of 3, 13, and 15%, respectively, at irrigation intervals of 2, 4, and 6 days, respectively. Additionally, at 0.5 (wt%) AS, root weight increased by 8, 15, and 16% in 2-, 4-, and 6-day irrigation intervals, respectively. Also, the height of the plant increased by 3, 5, and 17% at 2-, 4-, and 6-day irrigation intervals when 0.5 (wt%) of AS was used compared to the control. As well, essential oil percentage increased by 2.14, 2.06, and 1.63% at 2-, 4-, and 6-day irrigation intervals. The nutrient and protein contents decreased as irrigation intervals and AS usage increased, indicating a similar trend. However, compared with the control, the addition of 0.5 (wt%) of AS resulted in some improvements in nutrients and protein. The highest WUE (3.075 kg m^−3^) was attained in the 4-day irrigation interval and 1 wt% AS addition. This was followed closely by the 2-day irrigation interval with 1 wt% AS addition at 3.025 kg m^−3^, and the 4-day irrigation interval with 0.5 wt% AS addition, which reached 2.941 kg m^−3^. Overall, the use of AS in appropriate amounts (0.5 wt%) can reduce water consumption and enhance essential oil yield and WUE in peppermint cultivation in water-scarce arid and semi-arid regions.

## Introduction

There has been a significant increase in the cultivation and use of herbal medicinal and aromatic plants as a result of their pharmaceutical and therapeutic properties in recent years^1^. As a member of the Lamiaceae family, peppermint (*Mentha piperita* L.) is a natural hybrid between *Mentha aquatica* L. and *Mentha spicata* L.^2^. In addition to its medicinal and flavoring properties, peppermint is widely used in cosmetics, pharmaceuticals, and perfumery. Peppermint leaves contain glandular trichomes that store peppermint essential oil, which has a variety of environmental factors that affect its production including drought stress. Peppermint is sensitive to drought stress. Due to its spreading roots, it is not able to penetrate deep into the soil and becomes stressed due to drought stress. It has also been demonstrated that excess moisture in the soil negatively impacts its growth.

The drought stress problem is being addressed in many ways, including by using tolerant plants, and chemical, physical, hormonal, and biological methods. Under water scarcity, both fresh and dry matter, the yield of essential oils, and nutrient content are negatively affected. In Iran, the average rainfall is 250 mm per year, which is 70% less than the global average. Based on the type of plant, the severity, and the duration of drought stress, the productivity of the plants was reduced by an average of 13 to 94% under drought stress conditions^3^. Drought stress causes the reduction of photosynthetic rates, imbalances in the amount of water absorbed and lost, reactive oxygen species, and the closing of stomata. Furthermore, reactive oxygen species (ROS) are responsible for damaging the cells as a result of drought stress, which eventually leads to higher production of antioxidants in order to resolve this issue. Antioxidants in organisms enable them to cope with the oxidative stress produced by free radical damage. The majority of natural antioxidants are derived from plants. They are mainly composed of phenolic compounds, ascorbic acid, and carotenoids. It is therefore becoming increasingly critical to gain an understanding of the power of antioxidants derived from plants and to utilize their potential.

The stable free radical 2,2-diphenyl-1-picrylhydrazyl (DPPH) is a method that can be applied^4^. Therefore, it is critical to be able to cultivate these plants in different environments, especially in arid and semi-arid regions without sufficient rainfall. Also, this is an extremely crucial issue for the improvement of water use efficiency (WUE), and the use of advanced techniques in the agricultural sector. Several materials can be used to conserve soil moisture, such as organic residuals from plants, straws, plants, and animal manure, as well as synthetic materials such as superabsorbent polymers (SAPs). Due to their hydrophilic (-OH, -SO3H, -NH2), cross-linked macromolecular networks, and high porosity function, SAPs are able to absorb large amounts of liquid and retain them for long periods^5^. SAP absorbs and retains water through irrigation (artificial irrigation or rainwater) being applied to cultivated areas. In this way, the amount of water lost due to drainage and evaporation is reduced. As the soil dries, SAP releases the stored water in a controlled way through diffusion. This allows the soil to remain moist for prolonged periods of time. Furthermore, combining SAP with soil increases the size of the SAP. This increases soil porosity, which improves the supply of oxygen to the roots of the plants^5^.

Using SAP in arid and semi-arid areas improves soil texture, water retention in the plant growth environment, and irrigation efficiency. They can increase water productivity, reduce erosion, facilitate germination, and promote faster plant growth^6^. SAP may be used to increase crop water and fertilizer use efficiency mainly in arid and semi-arid regions where water management is an area of considerable research. Drought stress or limited water availability causes plants to suffer from oxidative stress and increased lipid peroxidation. Reduced height, reduced leaf area, and damaged leaf matrix are among the visible consequences. According to Essawy et al.^7^, a novel hydrogel polymer called CTS, derived from chitosan, increases the water-holding capacity of soil and triggers the release of nutrients. In addition to retaining plant nutrients, SAP can also facilitate their slow release to promote crop growth^8^.

According to the results of the analysis of five different samples of hydrogel polymers, these polymers enhance water retention in soil and reduce the evaporation of water from the soil by adding these materials, especially in hydrogels with larger particle sizes^9^. Beiranvandi et al.^10^ conducted a study to assess the impact of biochar and superabsorbent application on phytochemical parameters of *Satureja rechingeri* Jamzad under water stress. They investigated two biochar levels (0 and 10 t ha^−1^) and two Stockosorb superabsorbent levels (0 and 60 mg per plant) in various irrigation strategies. The results indicated that using both soil amendments reduced drought stress effects, increased growth rates, and enhanced the quantity and quality of *S. rechingeri* essential oil. Satriani, et al.^11^ examined the effect of cellulose hydrogel polymer application on bean cultivation under drought stress conditions in southern Italy. The polymer was mixed with soil in amounts of 0, 5, and 10 g. The results of this research showed that the efficiency index of WUE increased significantly with the use of polymer compared to the control treatment.

Situ et al.^12^ investigated the impact of varying doses of three superabsorbent polymers on soybean (*Glycine max* (Linn.) Merr.), and corn (*Zea mays* L.), growth, root morphology, and nutrient uptake. They found that while plant roots could directly absorb water by interacting with the hydrogel, the effect of superabsorbents on moisture-related factors affecting plant growth was minimal in conditions without drought stress and constant soil water potential. In contrast, applying a small amount of superabsorbent (0.2 wt%) improved soil physical properties, increasing soil porosity due to hydrogel swelling after water absorption. This enhanced root growth space, ultimately benefiting plant growth. The research results of de Vasconcelos et al.^13^ showed that the use of a new hydrogel composite based on starch significantly improves soil conditions for plant growth. They reported that the use of small amounts of hydrogel (less than 0.75 wt%) increased the growth of watermelon roots and generally enhanced the growth process of the plant in both clay and sandy soil. Research on a new biodegradable hydrogel called SAH in India revealed that this hydrogel has the ability to absorb up to 600 mL g^−1^ of water. It also improved the ability to retain water in the soil by 35%. Also, the use of this polymer increased soil porosity by 7% and improved chickpea growth^14^.

Zheng et al.^8^ conducted a comprehensive meta-analysis by analyzing 1504 data points from 310 articles (published before 2023) to assess the impact of SAP on crop yield and WUE across various treatments. This study covered several plant species, including maize (n = 446), oat (n = 16), cotton (n = 70), wheat (n = 402), vegetables (n = 156), oil crops (n = 99), and tuber crops (n = 314). The findings revealed that adding SAP to the soil resulted in an average increase of 12.8% in yields and 17.2% in WUE. While the general trend was positive, the magnitude of improvement varied among crop types, likely due to differences in water demand patterns and SAP functionality. The vegetable group exhibited the most significant increase in yield, while the tuber group saw the greatest improvement in the WUE parameter.

SAPs have been evaluated in several studies to determine how they affect plant yield. However, it is important to note that the capability of absorbing and storing water of these hydrophilic polymers was different due to their different synthesis and chemical structure. Therefore, their impact on the change in the moisture content of soils and their water retention will differ significantly. Moreover, the composition and amount of soil or irrigation water, the amount of clay in various soil textures, and the type of plant are also effective in evaluating the efficiency of a SAP polymer^9^. Aquasource superabsorbent (AS) is a new generation of hydrogel polymers based on potassium being high ecological standards. So far there have been no comprehensive studies on the effectiveness of this polymer in the water/soil/plant system. To maximize the potential of moisture-absorbent polymers, their efficiency should be assessed across various irrigation methods. However, excessive use of these polymers can negatively impact the growth environment and plant development. Therefore, determining the appropriate amount of moisture-absorbent polymer usage is crucial. Thus, this research investigates the effect of using different levels of AS on the quantitative and qualitative characteristics and WUE of peppermint at three irrigation managements (2, 4, and 6-day irrigation intervals).

## Materials and methods

### Experimental design and treatments

The experiment was conducted in 2018 at the research greenhouse of the Agricultural Engineering Research Institute (AERI) in Karaj city at latitude 35° 48' north and longitude 50° 58' east in order to evaluate the effects of AS on morphological characteristics, phytochemical properties, antioxidant content and WUE of peppermint. A factorial design with 3 replications was conducted. The two factors used in the statistical design were: (1) irrigation managements that have 2- (I2), 4- (I4), and 6-day (I6) irrigation intervals, and (2) AS/soil that has four weight percentages addition at zero (control), 0.5 (H0.5), 1 (H1), and 2 (H2) wt%. The main purpose of adding AS to the soil was to increase the water-holding capacity and increase irrigation intervals^15^. Different irrigation intervals were used to evaluate the amount of water released over time by the AS. This was done to determine how effective AS was at releasing water, and how the release rate could be adjusted to meet the needs of the plant. Thirty-six pots were filled using soil with a clay loam texture and specific amounts of AS (according to the stated weight percentages) were used in each pot before the sowing stage. To minimize light absorption competition, pots were placed at an appropriate distance from each other. Then the peppermint seedlings were transferred to the pots and due to the sensitive nature of the seedlings, irrigation managements were applied at the eight-leaf stage.

Seedlings of peppermint cultivar Black Mitcham, obtained from the Iranian Institute of Medicinal Plants, Karaj, Iran. The Institute of Medicinal Plants declared that peppermint seedlings were obtained under national and international guidelines, and the seedlings were prepared under the supervision and permission of the Agricultural Engineering Research Institute and all authors comply with all the local and national guidelines. The voucher specimens of the plants were deposited at the herbarium of the Iranian Institute of Medicinal Plants, Karaj, Iran. Before starting the studies in the greenhouse, the amount of moisture at the level of field capacity (FC) for the mixture of soil and different percentages of AS (0, 0.5, 1, and 2 wt% AS/soil) was determined using a pressure plate device. The amount of moisture at the field capacity level (FC) with the application of 0, 0.5, 1, and 2 wt% of polymer/soil was obtained as 20.62, 24.17, 25.80, and 31.70%, respectively. The amount of irrigation in each interval was determined based on the amount of moisture deficiency up to the field capacity. For this purpose, before each irrigation, the soil water content (SWC, cm^3^ cm^−3^) in each pot was measured using Lutron Professional Soil Moisture Meter (PMS-714). It should be noted that this device was calibrated prior to applying the treatments.

### Superabsorbent and soil properties

This research used a hydrogel polymer (AS brand) consisting of white granules of 1 μm–3 mm. AS is sterile, non-toxic, and climatically stable. This polymer is based on potassium, contains no acrylamide compounds, and is biodegradable. After the stability period has expired, the polymer decomposes into water, carbon dioxide, ammonia, and potassium compounds beneficial for plants. The AS’s swelling index in tap water is 1:400 (g/g), and its density is 0.5 (g cm^−3^). The soil used in this study had a clay loam texture (25.98% sand, 44.66% silt, 29.36% clay) with a bulk density of 1.4 (g cm^−3^). The bare soil’s field capacity, wilting point, and available water were 20.62, 9.07, and 11.55 (V%), respectively. The soil pH and EC were 7.01 and 2.12 (dS/m), respectively. The studied soil contained 8.2, 10.35, and 3.8 (meq/lit) of Na^+^, Ca^2+^, and Mg^2+^, respectively, and 8.17, 6.25, and 5 (meq/lit) of SO4^−2^, CL^−1^, and HCO_3_^−1^, respectively.

### Measurements

#### Morphological traits, yield, and WUE

The yield and yield components were measured after the reproduction stage, including root length and weight, leaf weight, plant height, and leaf relative water content. Immediately after cutting the leaves, fresh weights were determined following cutting, and dry weights were determined after the leaves were dried in the oven for 48 h at 70 °C^16^. The WUE was calculated using the following formula:

1 $$WUE = \frac{Y}{W}$$

where WUE is water use efficiency (kg m^−3^), Y is leaf yield (kg), and W is irrigation water volume (m^3^).

#### Essential oil extraction

All chemicals and reagents used in this research were analytical grade provided by Merck and Sigma-Aldrich, Germany. Using a Clevenger-type apparatus for three hours, 40 g of dried peppermint leaves were hydro-distilled with 400 mL of water. Then peppermint essential oil (EO) was extracted from the hydro-distilled leaves. To determine the chemical analysis of the samples, sodium sulfate was used to dry them after EO extraction. The essential oil yield was calculated using the following equation^17^:

2 $$EO\%=\frac{\mathrm{EO \;extracted \;by \;hydro \;distillation }}{40 \;gr \;dried \;leaves}$$

#### Nutrient content

A Kjeldahl method was used to determine peppermint leaves’ nitrogen (N) content^18^. Using a muffle furnace, 1 gr of dried peppermint leaves was placed in a porcelain crucible to determine its phosphorus (P), and potassium (K) concentrations. To determine the levels of P and K in dried peppermint leaves, 1 gr of dried peppermint leaves was immersed in a porcelain crucible and then put into a muffle furnace. A muffle furnace was heated to 550 °C gradually over eight hours until white ash was formed. After the muffle furnace was cooled, the samples were dissolved in 5 mL of hydrochloric acid (HCL) 2N. The samples were filtered through filter paper (Whatman No. 42) after 15 min, brought to volume (50 mL), and allowed to stand for 30 min. K and P concentrations were determined using flame photometry (Jenway PFP7C) and the yellow color method (the colorimetric method by ammonium vanadate/molybdate) using spectrophotometers at 470 nm absorption spectra, respectively^19^.

#### DPPH radical scavenging content

DPPH scavenging assay (DPPH SA) was conducted based on the modified approach of^20^. To obtain a final volume of 3 mL, 1 mL of 90 µM DPPH solution was mixed with different concentrations of each extract and methanol (95% v/v). The mixture was shaken immediately and allowed to stand for 30 min in the dark at room temperature. A colorimetric absorbance test was performed at 517 nm against a blank (the same solution without extracts). The radical scavenging capacity (RSC) was calculated using the following equation:

3 $$RSC\% = \left[ {\frac{Ablank - Asample}{{Ablank}}} \right] \times 100$$

where *A*_*blan*k_ was the absorption of the blank sample (t = 0 min) and *A*_*sample*_ was the absorption of the tested extract solution (t = 15 min).

#### Relative water content (RWC) and leaf area index (LAI)

First, the fresh weight of ten fully developed leaves (LFW) was measured to determine RWC in peppermint leaves. Leaf turgor weight (LTW) was measured within 24 h after the samples were kept in distilled water at 4 °C. Leaf dry weight (LDW) was determined after the leaves were dried in the oven at 70 °C for 48 h^16^. The following formula was used to calculate the RWC content:

4 $$RWC\% = \frac{LFW - LDW}{{LTW - LDW}} \times 100$$

LAI is determined by Delta-T area meter; Delta-T Devices Ltd., Cambridge, UK.

#### Chlorophyll and carotenoid

Half (0.5) g of peppermint fresh leaves were digested in liquid nitrogen and combined with 80% acetone to quantify the chlorophyll a (Ch_a_), b (Ch_b_), and carotenoid content. After centrifugation at 10,000 for 10 min, the supernatant was transferred to another tube, and the absorbance was measured using a spectrophotometer (UV-1800, Shimadzu, Japan). Afterwards, the following equations were used to determine the content of photosynthetic pigments^21^.

5 $$Ch_{a} = \left[ {\left( {12.7 \times Abs_{663} } \right) - \left( {2.69 \times Abs_{645} } \right)} \right]$$

6 $$Ch_{b} = \left[ {\left( {21.5 \times Abs_{645} } \right) - \left( {5.1 \times Abs_{663} } \right)} \right]Carotenoid$$

7 $$Carotenoid = \left[ {\left( {1000 \times Abs_{470} } \right) - \left( {1.82 \times Ch_{a} - 8502 \times Ch_{b} } \right)/198} \right]$$

#### Proline

First, 0.5 g of fresh peppermint leaves were digested in liquid nitrogen, combined with 10 mL of 3% sulfosalicylic acid, and centrifuged at 12,000 rpm. In a tube, 2 mL of the separated supernatant and samples were mixed with 2 mL of acid-ninhydrin and 2 mL of glacial acetic acid after 10 min. A water bath at 100 °C was used to heat the mixtures, and then they were cooled in an ice bath. Following this, 4 mL of toluene was added to the samples and vortexed for 20 s. As a final step, absorbance was measured spectrophotometrically at 520 nm and proline content was calculated as mol g^−1^ of fresh weight^22^.

#### Determination of protein, total phenolic, and flavonoid content

Using Bradford^23^, Singleton and Rossi^24^, and Nagy and Grancai^25^ methods, the protein, total phenolic, and flavonoid contents of peppermint were determined.

#### Statistical analysis

The main and interaction effects of irrigation interval (3 levels: 2, 4, and 6 days) and AS level (4 levels: 0, 0.5, 1, and 2 wt%) on morphological traits (root length, plant height, root dry weight, leaf dry weight), WUE, EO, nutrient content (nitrogen, phosphorus, and potassium), DPPH SA, RWC, LAI, chlorophyll (chlorophyll a, chlorophyll b, and chlorophyll total), carotenoid, proline, protein, total phenolic, and total flavonoid were determined using a 3 × 4 factorial design with 3 replications. The statistical analysis was done using the GLM procedure of SAS 9.4^26^. For each response variable, the validity of model assumptions was verified by examining the residuals as described in Montgomery^27^. For significant (*p* < 0.05) main effects, multiple means comparison was conducted using the lsmeans statement of Proc GLM at the 5% level of significance. However, when the interaction effect is significant, multiple means comparison was conducted at the 1% level of significance to reduce the over inflation of experiment-wise error rate due to the relatively large (3 × 4 = 12) means to compare.

## Results and discussion

### Morphological properties, essential oil percentage, and WUE

**Figure 1:**
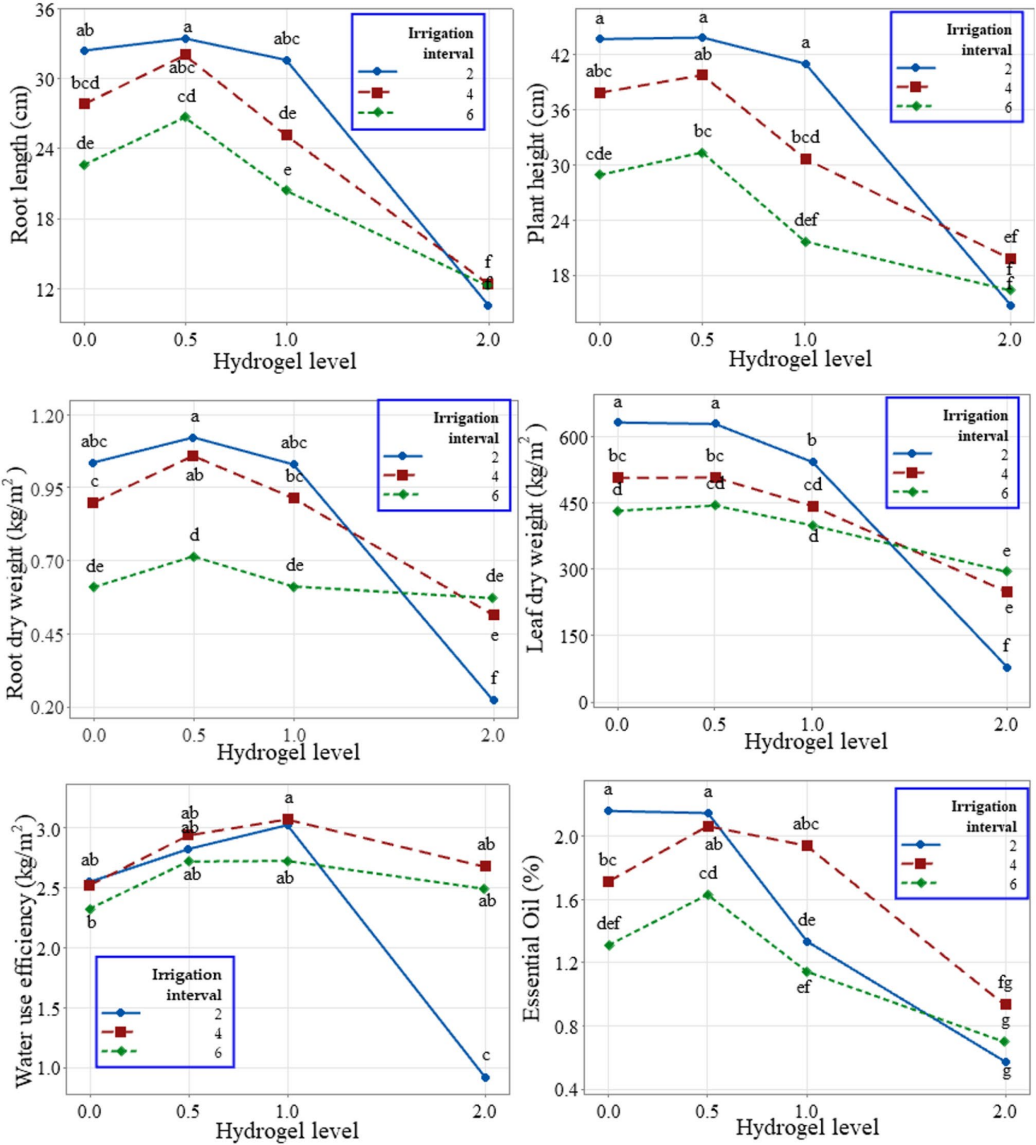
Interaction plot of root length (cm), plant height (cm), root dry weight (kg m^−2^), leaf dry weight (kg m^−2^), water use efficiency (WUE, kg m^−3^), and essential oil (EO, %) showing the means of the twelve combinations of irrigation intervals and hydrogel levels.

AS had a significant impact on root growth during the longer irrigation intervals (Table 1). The highest root length and weight were associated with an irrigation interval of 2 days (I2) and AS of 0.5 wt% (H0.5; Fig. 1). I2 and AS of 2 wt% (H2) were associated with the lowest root length and root weight. At H0.5, root length grew by 3, 13, and 15%, at irrigation intervals of 2, 4 (I4), and 6 (I6) days, respectively. In addition, root weight increased on I2, I4, and I6 at H0.5 by 8, 15, and 16%, respectively. Therefore, the application of AS at a level of 0.5 wt% resulted in an increase in root length and weight in comparison to the control (H0) treatment. However, increasing the AS level to 2% resulted in a significant decrease in root growth during all studied irrigation intervals. At high levels, AS absorbed a significant amount of water. However, this water content was not able to be used by the plant and created an unsuitable environment for root growth. At shorter irrigation intervals, this issue was particularly evident. But, at longer irrigation intervals, sufficient time was provided for water evaporation and soil moisture reduction, resulting in better root growth conditions.

**Table 1:** ANOVA *p*-values that show the significance of the main and interaction effects of irrigation and hydrogel levels on root length, plant height, root dry weight, leaf dry weight, water use efficiency (WUE), and essential oil (EO).

Source of variation	Root length	Plant height	Root dry weight	Leaf dry weight	WUE	EO
Irrigation	0.001	0.001	0.001	0.001	0.001	0.001
Hydrogel	0.001	0.001	0.001	0.001	0.001	0.001
Irrigation * Hydrogel	**0.003**	**0.007**	**0.001**	**0.001**	**0.001**	**0.001**
$\sqrt{MSE}$	2.373	4.069	0.066	31.32	0.278	0.166

$\sqrt{MSE}$ = the square root of the mean squares error (MSE), which estimates the common standard deviation (*σ*).

Significant effects that require multiple means comparison are shown in bold.

Plant roots are not only determined by genetic factors but also by the environment in which they grow. In the absence of available water, more photosynthetic products are allocated to the roots than to the stems, and closing the stomata results in a decrease in vegetative growth and an increase in root growth. Hydrogel polymers were reported to increase root length in sugar beets^28^. Due to its expansion and contraction, the SAP improved the soil's physical characteristics and porosity. Previous studies by Zheng et al.^8^ have shown that SAP can enhance soil porosity and aggregate formation by increasing soil particle cohesion and regulating shrinkage and swelling. However, a study conducted by Situ et al.^12^ showed that when a high amount of superabsorbent polymer was applied (e.g., 0.6 wt%), the promotion of growth by the superabsorbent gets countered by its negative impact on root growth, resulting in an overall reduction in root biomass.

The effects of AS levels and irrigation intervals on plant height showed a similar trend to that of root length. Based on the comparison of the means related to irrigation interval and AS levels (Fig. 1), it appears that increasing the irrigation interval from 2 to 4 days had no significant effect on the height of peppermint plants. Thus, increasing the irrigation interval to 6 days resulted in a reduction in the height of the plants. In comparison to the control treatment, the use of 0.5 wt% AS at I6 resulted in a significant increase in plant height. The plant height in the I2 irrigation management did not differ significantly from the control treatment and when AS was applied at 1 wt% (H1). However, a decrease in plant height was observed when the amount of AS was increased by up to 2%. At the level of H0.5, compared to the control treatment in each irrigation management, the plant height increased by 3%, 5%, and 17%, in I2, I4, and I6, respectively. With an irrigation interval of 2 days (using 0.5 (wt%) of AS, H0.5), the highest plant height was 44.8 cm. Additionally, the lowest plant height of 14.7 cm was achieved using (2%wt) AS and a 2-day irrigation interval. As shown in Fig. 1, increasing the irrigation intervals, especially in the control treatment, caused a decrease in plant growth characteristics due to drought stress conditions.

Under low water supply, cell elongation was reduced^29^. Plant height was reduced by drought stress due to a reduction in turgidity, growth, volume, and the number of stem cells^30,31^. Consequently, a shortening of the internode distance caused a reduction in plant height^29^. As mentioned before, the application of AS at the rate of 0.5 wt% in all irrigation managements improved the growth characteristics of peppermint compared to the control treatments. Yang et al.^32^, Keshavars et al.^33^, Jahan et al.^34^, and Mohebi^35^ have reported a significant effect of superabsorbent on the elongation of stems. Due to the superabsorbent’s high capacity for absorbing and storing water in the soil, this phenomenon occurred. As a result of the addition of superabsorbent polymers to soil, the physical and structural properties of soil were modified^36,37^.

As shown in Fig. 1, in the case of leaf yield, the control treatment and H0.5 were in the same statistical group under all applied irrigation managements. However, yield reduction was observed at the levels of H1 and H2 in comparison to the control treatment in I2, I4, and I6. These treatments did not provide optimal growth conditions for peppermint. So, in H1 and H2, despite absorbing moisture, AS has not been able to effectively release water gradually. Due to the decrease in available water, the reduction of leaf area led to a reduction in the transfer of photosynthetic materials to the flowers. Therefore, the dry weight of the leaf per unit area was reduced, and this caused a decrease in the production of photosynthetic substances^38^.

Zheng et al.^8^ found that SAPs increased crop yields, but the degree of increase varied significantly among crop types. Yield improvements ranged from 9.3% to 18.3% across six categories, with vegetables showing the highest enhancement (18.3%), followed by tubers (17.7%), oils (15.2%), oats (11.5%), wheat (10.4%), maize (9.6%), and cotton (9.3%). SAPs efficiently absorb and gradually release nutrients, reducing fertilizer losses and increasing nitrogen use efficiency, both contributing to higher yields due to the gradual nutrient release. Abedi-Koupai and Asadkazemi^39^ showed that the use of SAPs enhanced greenhouse cucumber characteristics. As a result of this research, there has also been a significant improvement in some growth parameters using AS at H0.5 and H1. This indicates that AS has mitigated the negative effects of reduced water availability. However, the application of higher amounts (H2) led to a significant decrease in growth parameters. Therefore, a decrease in yield and yield components was caused by unfavorable conditions, especially root growth.

The AS caused the stomata to remain open for a long period, resulting in the correct stabilization of carbon dioxide and increased plant yield^33^. According to Abedi-Koupai and Asadkazemi^39^, the optimal amount of Super Water A-200 polymer for a greenhouse cucumber production system was 4 (g kg^−1^) of soil. Additionally, increasing the amount of AS consumption to 6 (g kg^−1^) will result in a decrease in yield due to a reduction in soil ventilation. The results of research on cotton^40^, and wheat^41^ showed that drought stress during the growth period of these two plants led to an increase in WUE. Also, according to Alishah et al.^42^, cucumber yield decreased when the level of AS was increased from 2 to 4 and 8 (g kg^−1^) of soil. This issue was attributed to the high absorption of water by the superabsorbent polymer. As a result, the soil cooled and plant growth was adversely affected.

According to Sreevalli et al.^43^, yield reduction during increased drought stress can be related to the increase in the allocation of photosynthetic materials to the root compared to the aerial part of the plant, which was similar to the results of this study. Martins Melo et al.^44^ found that applying superabsorbent polymer (Ca-SAP) increases plant-available water by an average of 0.16 m^3^ m^−3^ compared to the control (without superabsorbent). This increase in available water ultimately improves WUE while maintaining lettuce yield compared to the control treatment. In terms of essential oil percentage, a decrease in the percentage of this parameter was observed after increasing the irrigation interval to 6 days and increasing the level of AS up to 2% (H2). Based on an analysis of the mutual effect of AS levels and irrigation intervals (Fig. 1), the use of this polymer up to 0.5% (H0.5) improved essential oil production in all treatments. With the increase of drought stress intensity from I2 to I4 and I6, the role of superabsorbent became more obvious and the essential oils improved significantly. As a result of applying different irrigation managements, I2, I4, and I6 obtained the most essential oil levels by 2.14, 2.06, and 1.63%, respectively, in H0.5.

Using AS increased the percentage of essential oil in I4 and I6 (drought stress) by 20 and 24%, respectively, compared with the control treatment. The use of higher amounts of AS (1 and 2 wt%) resulted in a decrease in EO values, so that the lowest EO values were obtained in H2 in all studied irrigation intervals. A study by Beiranvandi et al.^10^ indicated that Stockosorb superabsorbent increases water absorption and storage in the soil, improves nutrient storage, enhances soil structure, and nutrient uptake. They reported that Stockosorb improved the growth, yield, and essential oil quality of *Satureja rechingeri* Jamzad, leading to a 6.34% increase in essential oil compared to the control. As shown in Fig. 1, WUE was affected by different irrigation managements and the application of different amounts of the AS. Since the highest WUE was obtained in I4 (at all AS levels), increasing irrigation intervals improved WUE due to reduced water consumption.

High WUE was associated with the addition of 1 wt% of AS in all examined irrigation intervals. Increasing AS usage to 2 wt% in all intervals led to a reduction in WUE. Excessive AS usage caused soil saturation and poor ventilation, creating unfavorable conditions for plant growth. The largest decline in WUE occurred in I2, where the short irrigation interval hindered proper moisture drainage from the AS, negatively impacting soil ventilation. The highest WUE values were observed during 4-day irrigation intervals. Overall, maximum WUE (3.375 kg m^−3^) was achieved during the 4-day irrigation interval with 1 wt% AS, followed by the 2-day interval with 1 wt% AS, and then the 4-day interval with 0.5 wt% AS at 3.025 and 2.941 (kg m^−3^), respectively.

Drought stress affects WUE differently depending on the plant species, its phenological stage, and the duration and intensity of the stress. It has been reported that as a result of SAP swelling, gravitational water can be converted into plant-available water and thus improves water use efficiency^45^. These improvements in WUE are associated with increased water-stable aggregates, soil porosity, organic matter, and the availability of nitrogen, phosphorus, and potassium post-SAP application. Application of SAP significantly increased water-stable aggregates (by 18.9%) and total soil porosity (by 18.0%). Furthermore, SAP had a substantial impact on soil chemical properties (*p* < 0.05), leading to a 7.5% rise in soil organic matter, 6.0% more available nitrogen, 7.3% extra available phosphorus, 5.4% increased potassium, and an 8.73% rise in total nitrogen content.

It has been reported that the WUE of greenhouse cucumber increased with the use of SAPs^39^. The results of this study were in accordance with those reported by Keshavars et al.^33^, and Jahan et al.^34^. But it is very important to determine the optimal amount of super absorbent to achieve the maximum WUE. In this study, increasing the AS consumption to 2% caused a decrease in WUE. Abedi-Koupai and Asadkazemi^39^ also stated that the highest WUE in greenhouse cucumber production was achieved at the level of 4 g of superabsorbent per kilogram of soil, and with a further increase in the amount of superabsorbent, the WUE decreased. The research findings of Takahashi et al.^45^ showed that low rates of SAP application are more suitable for maximizing water available to plants. According to the results, using AS of 0.5 (wt%) led to increased WUE. However, with increasing AS level by up to 2%, WUE decreased. Despite the use of less water at higher levels of AS, the plant was not able to absorb soil moisture. In these treatments, the decrease in yield resulted in a decrease in WUE. Due to improper ventilation and a decrease in soil temperature, high soil moisture created unfavorable conditions for plant growth in these treatments.

### Nutrient and protein content

According to the analysis of variance results (Table 2), the values of N, P, and K were significantly affected by irrigation intervals and AS amounts, as well as the interaction of these two factors. The results shown in Table 3 indicate that the highest amount of N, P, and K was obtained in I2H0.5. Regarding the application of different levels of AS, the use of 0.5% superabsorbent increased the amount of N, P, and K by about 2, 7, and 3%, respectively, compared to the control treatment in I2. Meanwhile, the highest contents of N, P, and K in peppermint from I2H0.5 were 27.0, 2.15, and 28.7 (g kg^−1^), respectively. Also, I2H2 had the lowest N, P, and K contents (14.4, 1.07, and 16.0 [g kg^−1^], respectively; Table 3).

**Table 2:** ANOVA *p*-values that show the significance of the main effects and interaction effect of irrigation and hydrogel levels on nitrogen (g kg^−1^), phosphorus (g kg^−1^), potassium (g kg^−1^), proline (mmol g^−1^ fresh weight), and protein (%).

Source of variation	Nitrogen	Phosphorus	Potassium	Proline	Protein
Irrigation	0.001	0.001	0.001	0.001	0.001
Hydrogel	0.001	0.001	0.001	0.001	0.001
Irrigation * Hydrogel	**0.001**	**0.001**	**0.001**	**0.001**	**0.001**
$\sqrt{MSE}$	1.259	0.141	1.650	0.091	0.347

$\sqrt{MSE}$ = the square root of the mean squares error (MSE), which estimates the common standard deviation (*σ*).

Significant effects that require multiple means comparison are shown in bold.

**Table 3:** Mean values of nitrogen (g kg^−1^), phosphorus (g kg^−1^), potassium (g kg^−1^), proline (mmol g^−1^ fresh weight), protein (%) obtained from the twelve combinations of irrigation interval (Irr) and hydrogel levels (Hydrogel).

Irr	Hydrogel	Nitrogen	Phosphorus	Potassium	Proline	Protein
2	0.0	26.3^a^	1.98^ab^	27.8^a^	0.630^g^	13.20^ab^
2	0.5	27.0^a^	2.15^a^	28.7^a^	0.520^g^	13.51^a^
2	1.0	25.0^ab^	1.71^bc^	25.1^ab^	0.867^f^	12.43^bc^
2	2.0	14.4^g^	1.07^f^	16.0^e^	1.640^cd^	8.86^g^
4	0.0	21.8^cd^	1.47^cde^	21.0^cd^	0.700^fg^	12.01^cd^
4	0.5	23.2^bc^	1.68^bc^	21.9^bc^	0.550^g^	12.31^c^
4	1.0	18.9^e^	1.51^cd^	19.0^cde^	1.310^e^	11.20^e^
4	2.0	15.9^fg^	1.15^ef^	17.0^e^	2.053^a^	10.21^f^
6	0.0	18.0^ef^	1.36^def^	18.6^cde^	1.573^d^	11.33^de^
6	0.5	19.1^de^	1.52^cd^	19.8^cde^	1.490^de^	11.71^cde^
6	1.0	17.0^efg^	1.32^def^	17.8^de^	1.817^bc^	10.18^f^
6	2.0	16.3^efg^	1.20^def^	17.3^de^	2.003^ab^	9.71^f^

Means in each column followed by the same letter are not significantly different at the 5% level of significance.

Increasing the irrigation interval, which reduces the available water for the plant, leads to decreased nutrient absorption by peppermint. The addition of AS to the soil mitigates these negative effects by improving nutrient absorption through initial nutrient uptake and gradual release. Raza et al.^46^ found that drought stress reduces wheat’s absorption of essential nutrients (NPK), but applying biochar as a soil conditioner significantly mitigates these effects. SAP materials effectively reduced fertilizer losses and regulated nutrient release, enhancing soil nutrient content. Under SAP application, larger macro-aggregates in soils lead to increased stability and more efficient nutrient storage. Additionally, SAP application increased soil nitrogen, nitrogen availability, phosphorus availability, and potassium availability, supporting nutrient needs during plant growth^8^. The analysis of variance results (Table 2) showed a highly significant interaction effect of these two factors on the protein content of peppermint. Based on the results of the multiple means comparison, the highest amount of protein was obtained in the control treatment and I2H0.5. The lowest value of this trait was related to H2 in two irrigation managements I4 and I6, as well as H1 in irrigation management I6. In general, it can be concluded that the amount of protein was reduced by increasing the irrigation intervals and by increasing the percentage of AS usage. The application of AS at a low level in H0.5 led to some improvement in the protein compared to the control treatment in all irrigation interval treatments. By reducing total soluble protein in plants, drought stress disrupts the protein synthesis process, resulting in a decrease in protein production and a rise in protein degradation^47^.

The reduction of phosphorus in soil under drought stress was related to the reduction of soil water content. This caused a reduction in the transfer of nutrients from the soil to the plant. Due to the strong absorption of phosphorus by clay particles in the soil, phosphorus is one of the ions that plants cannot utilize under water-stress conditions. Only a small amount of P is present in the soil as a solution. Amirnia et al.^48^ reported that under drought stress conditions, the absorption of P by plants was reduced due to its low solubility and the reduction of its absorption ability by the plant roots. Soil phosphorus absorption was accelerated and improved by the use of a super-absorbent hydrogel. This was due to improving soil ventilation, preventing leaching, and increasing the solubility of soil mineral elements. The increase in nutrient absorption due to organic fertilizers could therefore be explained by the morphological changes in the roots of the plants, particularly the increase in the length, number, and thickness of the roots. Under drought stress, potassium levels decreased due to a reduction in soil water, which in turn resulted in a reduction in plant mineral uptake from the soil^48^.

Due to the inability of AS to release absorbed moisture, high levels of this polymer (1 and 2 wt%) create competition between the polymer and the plant roots for the ability to absorb moisture, resulting in drought stress. Since SAPs were effective at stabilizing soil particles, the use of superabsorbent hydrogels in small amounts improved soil hydraulic conductivity. In consequence, this resulted in the development and expansion of the root system of the plant, as well as an improved ability to absorb water and nutrients.

By providing the moisture and nutrients required by the plant in the treatment of low levels of AS (H0.5), a suitable environment for root growth and absorption of nutrients was provided for the plant, which caused an increase in the percentage of nitrogen^49^. Moreover, in water-deficient conditions, microbial activity in the soil decreased nutrient solubility and availability^50^. Salehi et al.^51^ observed that in chamomile (*Matricaria chamomilla* L.), concentrations of nitrogen, phosphorus, and potassium decreased under conditions of drought stress. Hence, the high protein content with small amounts of the hydrogel can be attributed to the faster absorption of nitrogen by the plant and the increase in nitrogen concentration in the aerial parts. Nitrogen plays an effective role in the structure of chlorophyll and is the most significant mineral element in the synthesis of proteins, and its increase under favorable conditions led to an increase in the amount of protein^48^.

### Proline content

As a result of reactive oxygen species compounds enhancing membrane lipid peroxidation and releasing a range of aldehydes, proline was found to be increased under mild and severe drought stress conditions (I4H2 and I6H2)^52,53^. Therefore, the osmotic potential of the plant cells was reduced by the increase in osmotolerant metabolites, and the plant cells' turgescence pressure was maintained. Consequently, reactive oxygen species compounds and lipid peroxidation were reduced, and membrane stabilization was enhanced^54^.

Under moderate and severe drought stress conditions, Javanmard et al.^55^ observed an increase in proline content in *Lallemantia Iberica*. Since the lowest amounts of proline in different irrigation interval treatments were obtained in the treatment of 0.5% AS application, it can be concluded that the application of this amount of AS caused the adjustment of drought stress conditions and improved plant growth conditions (Table 3). As proline is an amino acid compound, and nitrogen is an essential element in the amino acid structure, the higher proline concentration achieved by applying superabsorbent may be attributed to increased availability of nitrogen^56^.

### Chlorophylls content

Analysis of variance results showed that the effects of irrigation interval and AS amount on chlorophyll a, chlorophyll b, and total chlorophyll were significant at the 1% level (Table 4). As shown in Table 5, the highest value of all three chlorophylls was obtained in I2H0.5. Increasing the amount of AS to H2 resulted in a significant decrease in chlorophyll. The amount of total chlorophyll in H0.5 increased by 1.8, 15, and 44% in I2, I4, and I6, respectively, compared to the control treatment. However, by increasing the amount of AS from H1 to H2 and creating unsuitable conditions for plant growth, total chlorophyll decreased by 77, 55, and 13%, in I2, I4, and I6, respectively. Similar trends were observed in chlorophyll a and chlorophyll b. Thus, increasing the amount of AS could compensate for the adverse effects of drought. While, at all AS levels (H0, H0.5, H1, and H2), the amount of chlorophyll a, chlorophyll b, and total chlorophyll contents decreased with increasing irrigation intervals.

**Table 4:** ANOVA *p*-values that show the significance of the main effects and interaction effect of irrigation and hydrogel on chlorophyll a (mg g^−1^ fresh weight), chlorophyll b (mg g^−1^ fresh weight), chlorophyll total (mg g^−1^ fresh weight), relative water content (RWC, %), and leaf area index.

Source of variation	Chlorophyll a	Chlorophyll b	Chlorophyll total	RWC	Leaf area index
Irrigation	0.001	0.001	0.001	0.001	0.001
Hydrogel	0.001	0.001	0.001	0.001	0.001
Irrigation * Hydrogel	**0.001**	**0.001**	**0.001**	**0.001**	**0.001**
$\sqrt{MSE}$	0.178	0.088	0.112	4.187	0.225

$\sqrt{MSE}$ = the square root of the mean squares error (MSE), which estimates the common standard deviation (*σ*).

Significant effects that require multiple means comparison are shown in bold.

**Table 5:** Mean values of chlorophyll a (mg g^−1^ fresh weight), chlorophyll b (mg g^−1^ fresh weight), chlorophyll total (mg g^−1^ fresh weight), relative water content (RWC, %), and leaf area index obtained from the twelve combinations of irrigation interval (Irr) and hydrogel levels (hydrogel).

Irr	Hydrogel	Chlorophyll a	Chlorophyll b	Chlorophyll total	RWC	Leaf area index
2	0.0	4.917^a^	1.657^a^	6.58^a^	67.4^a^	3.68^a^
2	0.5	5.010^a^	1.687^a^	6.70^a^	67.0^a^	3.65^a^
2	1.0	3.707^bc^	1.453^b^	5.16^b^	55.1^bc^	2.65^bc^
2	2.0	0.833^f^	0.323^e^	1.15^i^	31.0^f^	1.80^e^
4	0.0	3.543^c^	1.030^c^	4.57^c^	63.5^ab^	2.63^bc^
4	0.5	3.983^b^	1.280^b^	5.26^b^	65.7^a^	2.94^b^
4	1.0	2.133^e^	0.803^d^	2.93^e^	48.3^cd^	2.41^cd^
4	2.0	0.897^f^	0.393^e^	1.29^hi^	31.1^f^	1.85^e^
6	0.0	1.223^f^	0.753^d^	1.97^f^	41.2^de^	2.14^cde^
6	0.5	2.700^d^	0.853^cd^	3.55^d^	47.4^cd^	2.21^cde^
6	1.0	1.163^f^	0.483^e^	1.64^g^	44.6^d^	2.01^de^
6	2.0	0.980^f^	0.440^e^	1.42^gh^	32.8^ef^	1.88^e^

Within each column, means sharing the same letter are not significantly different.

When peppermint was exposed to mild and severe drought stress, its chlorophyll a, chlorophyll b, and total chlorophyll contents were reduced due to high ROS activity and lipid peroxidation^57^. As a result of increased photo-oxidation of chlorophyll as well as chloroplast breakdown, Jahani et al.^58^ found that peppermint's concentration of total chlorophyll was decreased by 9.1 and 31.4% under mild and severe drought stress conditions.

According to Aslam et al.^59^, drought stress has resulted in a decrease in chlorophyll levels in quinoa leaves. The drought stress disrupts the activity of the enzyme (chlorophyllase), reducing leaf chlorophyll and as a consequence reducing photosynthesis^60^. According to Zaheer et al.^61^, drought stress in the growth stages of wheat leads to a reduction in leaf chlorophyll, but the use of rhizobacteria and cytokinins decreases the negative effects of drought stress. According to Bhusal et al.^62^, chlorophyll content may be reduced under drought stress conditions as a result of stomata closing and chloroplast protein biosynthesis being inhibited. This study found that the application of superabsorbent enhanced chlorophyll content in drought-stressed plants. Plant chloroplasts contain chlorophyll consisting of a porphyrin head and a long hydrocarbon with a phytol sequence^63^. Porphyrin is composed of four nitrogen-containing pyrrole rings. It is a chelate formed in which a magnesium ion binds with four nitrogen atoms in the center of a chlorophyll molecule. The increase in chlorophyll content is likely due to the increased accessibility of nutrients provided by superabsorbents since chlorophylls correlate significantly with nutrients. A study conducted by Gholinezhad et al.^64^ showed the use of the appropriate amount of superabsorbent leads to an increase in chlorophyll.

### Relative water content and leaf area index

As shown in Table 4, different irrigation managements and different amounts of AS significantly affected RWC and LAI. The mutual effect of different irrigation managements and different amounts of AS for RWC and LAI shows that the highest amount of RWC, and LAI was obtained in I2H0.5 (Table 5). By increasing the amount of superabsorbent to 1 and 2%, unfavorable conditions were created for plant growth and these two response variables (RWC and LAI) were reduced.

In I2H0.5 when suitable conditions for plant growth have been provided, the control treatment had the highest values of RWC, and LAI at 67% and 3.65, respectively. By increasing the irrigation interval to 4 days, the highest values of RWC, and LAI were achieved to be 65.7% and 2.94, respectively in the H0.5 treatment. There was a similar trend in the irrigation interval of 6 days, and the highest values of both response variables were obtained at H0.5.

A plant’s RWC describes how much water was in its cells, especially under drought stress conditions^65^. During drought stress conditions, RWC decreased due to a decrease in root absorption and an increase in leaf transpiration. In mild drought stress conditions, peppermint's RWC content was relatively enhanced by superabsorbent application.

In a symbiotic relationship, arbuscular mycorrhizal fungi facilitate the flow of water into root cells, thereby increasing the amount of water in plant cells. Water moves across roots with less resistance as fungal hyphae penetrate the cortex and endoderm of roots. Consequently, there is less resistance in the xylem until the water reaches the root^66^. Gholinezhad et al.^64^ found that when grown under moderate conditions, sesame's RWC content decreased by 23%. According to Iqbal et al.^40^, drought stress impairs wheat's physiological traits. The application of micronutrients (iron and zinc) mitigates these effects by enhancing leaf osmotic potential, leaf water potential, and relative water content (RWC). Reduced turgidity is the initial sign of water deficiency, leading to diminished cell growth and development, particularly in stems and leaves. As cell growth slowed down, organ size was limited. This was why the first noticeable effect of dehydration on plants could be detected in reduced leaf area or plant height. In addition, in low water conditions, the absorption of nutrients was also reduced and therefore the growth and development of the leaves were limited. Following the reduction of leaf area, light absorption was also reduced, and the total photosynthetic rate in water shortage conditions, plant growth, and performance were reduced^32^.

### Carotenoid content

The analysis of variance results shown in Table 6 revealed significant effects of AS and irrigation managements. Table 7 shows that the amount of carotenoid was not statistically different in I2H0.5, I4H0.5, and I6H0.5, as well as in I2H2, I4H2, and I6H2. On the other hand, the highest carotenoid content was obtained in H1 when different amounts of irrigation intervals were applied. Therefore, the creation of mild stress conditions led to an increase in carotenoid content. Additionally, with an increase in irrigation intervals from 2 to 4 days and moderate stress conditions, the amount of carotenoid increased slightly. However, when the amount of stress was increased from I4H1 to I6H1 as well as from I4H2 to I6H2 severe stress conditions were created, and the carotenoid content decreased by 24 and 8%, respectively.

**Table 6:** ANOVA *p*-values that show the significance of the main effects and interaction effect of irrigation and hydrogel on carotenoid (mg g^−1^ fresh weight-FW), total phenol content (mg g^−1^), and 2,2-diphenyl-1-picrylhydrazyl scavenging assay (DPPH SA, %).

Source of variation	Carotenoid content	Total phenol content	Total flavonoids content	DPPH SA
Irrigation	0.008	0.001	**0.001**	0.001
Hydrogel	0.001	0.001	**0.001**	0.001
Irrigation*Hydrogel	**0.001**	**0.001**	0.873	**0.001**
$\sqrt{MSE}$	0.120	1.123	0.969	2.701

$\sqrt{MSE}$ = the square root of the mean squares error (MSE), which estimates the common standard deviation (*σ*).

Significant effects that require multiple means comparison are shown in bold.

**Table 7:** Mean values of carotenoid (mg g^−1^ fresh weight-FW), total phenol content (mg g^−1^), and 2,2-diphenyl-1-picrylhydrazyl scavenging assay (DPPH SA, %) obtained from the twelve combinations of irrigation interval (Irr) and hydrogel levels (hydrogel).

Irr	Hydrogel	Carotenoid	Total phenol content	DPPH SA
2	0.0	1.717^ab^	26.1^de^	37.6^bc^
2	0.5	1.570^bcd^	26.1^de^	36.8^bc^
2	1.0	1.657^bc^	31.7^a^	49.0^a^
2	2.0	0.987^g^	25.7^de^	27.2^d^
4	0.0	1.653^bc^	31.0^ab^	41.5^b^
4	0.5	1.420^cde^	30.9^ab^	40.4^b^
4	1.0	1.940^a^	32.0^a^	49.4^a^
4	2.0	1.130^fg^	26.2^cde^	35.5^bc^
6	0.0	1.320^def^	28.1^cd^	40.7^b^
6	0.5	1.470^bcde^	28.7^bc^	41.0^b^
6	1.0	1.457^bcde^	25.5^e^	32.0^cd^
6	2.0	1.233^efg^	25.2^e^	27.6^d^

Within each column, means sharing the same letter are not significantly different.

Under mild drought stress, carotenoid concentrations were increased. Both enzymatic and non-enzymatic antioxidant systems protect plant tissues from oxidative damage caused by drought stress. In plants under stressful conditions, non-enzymatic antioxidants such as carotenes (xanthophylls and carotenes), alpha-tocopherol, and ascorbates reduce ROS activity^67^. Therefore, under drought stress, the application of superabsorbents enhanced carotenoid content.

### Antioxidant content

As can be seen in Table 6, changing the irrigation managements and different levels of AS had a significant effect on the 2,2-diphenyl-1-picrylhydrazyl scavenging assay (DPPH SA, %). Table 7 shows the comparison of the mean values of DPPH SA percentage obtained from the twelve combinations of irrigation interval and AS levels. The results showed that the highest amount of DPPH SA (%) was observed in I2H1 and I4H1 treatments, which were 49 and 49.4%, respectively. On the other hand, by increasing the irrigation period to 6 days, the amount of DPPH SA (%) in I6H1 decreased by 34 and 35%, respectively, compared to I2H1 and I4H1. While in the H2 treatment, the highest amount of DPPH SA (%) was associated with the I4 treatment, and no significant difference was observed between I2H2 and I6H2.

As shown in Table 6, total phenolic content (TPC) was significantly affected by the interaction of irrigation managements and the amounts of AS. While total flavonoid content (TFC) was not significantly affected by this interaction. I4H1 gave the highest TPC, and by changing the irrigation management from 2 to 4 days in H1 and creating medium stress conditions, the amount of TPC increased from 31.7 to 32 mg/g (Table 7). However, more stress at an irrigation interval of 6 days resulted in a decrease in TPC.

Table 8 shows multiple means comparison results of the levels of irrigation and AS conducted due to the significance of their main effects on TFC. The results showed that the highest amount of TFC occurred in I4 and H1 (15.4 and 15.8 mg/g, respectively), which indicated medium stress. Severe stress in H2 reduced the values of TFC by 23% compared to that in H1. Therefore, Peppermint treated with 35 and 24% soil moisture, under water deficit conditions, showed increased antioxidant content, phenolic content, and flavonoid content compared to control plants. Similarly, another study on *Vitis vinifera* L. found that phenolic compounds were negatively affected by long-term drought stress^68^. Abiotic stresses, however, have been shown to enhance the production of phenolic compounds in plants. As a response to drought stress, plants produced antioxidant enzymes, followed by phenolic compounds, which served as non-enzymatic drought tolerance factors. Many environmental factors affect the biosynthesis of phenolic compounds derived from the shikimic acid pathway^69^. Plants are protected against stress by phenolic and flavonoid compounds.

**Table 8:** Mean values of total flavonoid (mg g^−1^) obtained from the three irrigation intervals (Irr) and the four hydrogel levels (hydrogel).

Irr	Total flavonoid	Hydrogel	Total flavonoid
2	13.5^b^	0.0	14.1^b^
4	15.4^a^	0.5	14.0^b^
6	13.1^b^	1.0	15.8^a^
		2.0	12.1^c^

Within each column, means sharing the same letter are not significantly different.

During stress, plants produce ROS, and it appears that the flavonoids, biosynthesis of antioxidants, and secondary metabolites contribute to the protection of plant cells from ROS detoxification, as well as improving protein and amino acid stabilization^62^. Plants grown in greenhouse conditions have higher concentrations of TPC and antioxidant content than plants grown in fields. The scavenging activity of Bunium persicum essential oil has been demonstrated to be enhanced by drought stresses^70^.

## Conclusions

Abiotic stresses, like water scarcity, have detrimental effects on agronomy, plant physiology, and production. Superabsorbent hydrogels can serve as valuable tools for effective water resource management and mitigating drought stress in plants. In this study, different irrigation methods and varying AS application levels influenced the phytochemical and morphological characteristics of peppermint. Drought stress had a negative impact on peppermint's morphological traits. However, the application of 0.5 wt% AS improved these characteristics and alleviated the effects of drought stress. This improvement was attributed to increased nutrient uptake, antioxidant content, chlorophyll production, leaf area index, and soil moisture. The highest essential oil yields, at rates of 2.14%, 2.06%, and 1.63%, were obtained using 0.5 wt% AS in the I2, I4, and I6 treatments, respectively. The highest AS usage resulted in the lowest water use efficiency (WUE), suggesting that while the polymer can absorb water, it may not release and make it readily available to plants at higher levels. Drought stress and the use of superabsorbent caused various metabolic and molecular changes in peppermint. Despite the reduction in morphological traits due to drought stress, it increased the antioxidant content of peppermint extract. In conclusion, the use of 0.5 wt% AS in regions with arid and semi-arid climates can reduce water consumption and enhance WUE. However, it is essential to determine the appropriate AS dosage based on the crop type and suitable irrigation management for optimal superabsorbent utilization.

## Data Availability

The datasets used or analyzed in the current study are available from the corresponding author upon a reasonable request.
